# An Online Observer for Minimization of Pulsating Torque in SMPM Motors

**DOI:** 10.1371/journal.pone.0153255

**Published:** 2016-04-18

**Authors:** Lucian Roșca, Mihai Duguleană

**Affiliations:** 1 Faculty of Engineering, University “Lucian Blaga” of Sibiu, Sibiu, Romania; 2 Department of Product Design and Robotics, University Transilvania of Brasov, Braşov, Romania; Chongqing University, CHINA

## Abstract

A persistent problem of surface mounted permanent magnet (SMPM) motors is the non-uniformity of the developed torque. Either the motor design or the motor control needs to be improved in order to minimize the periodic disturbances. This paper proposes a new control technique for reducing periodic disturbances in permanent magnet (PM) electro-mechanical actuators, by advancing a new observer/estimator paradigm. A recursive estimation algorithm is implemented for online control. The compensating signal is identified and added as feedback to the control signal of the servo motor. Compensation is evaluated for different values of the input signal, to show robustness of the proposed method.

## Introduction

Electro-mechanical drives are widely used in a large variety of industrial applications. Due to flat speed/torque characteristics, high dynamic response and good controllability, the permanent magnet (PM) motors are appealing candidates for high-performance applications where position accuracy is essential. However, a persistent problem with PM motors is the appearance of the pulsating torque, which further translates into shaft vibration, acoustic noise and damage to drive components. These phenomena usually appear at lower speeds [1], but are also present in other situations. Periodic disturbances responsible for this problem are defined as the sum of cogging torque, ripple torque and mechanical beating, resulting in vibration and noise unacceptable for high-performance positioning systems.

Torque smoothness is required in sensitive equipment ranging from industrial robots to auto parts such as electric-assisted power steering drives. That is why in the last decades, several studies have targeted this issue.

There are mainly 2 different ways of reducing the pulsating torque [2, 3]: by modifying the design of the motor or by actively controlling the motor using i.e. a self-tuning controller. The first category relies on the optimization of the structural parameters of the motor (shifting asymmetrically the magnets, pairing the rotor teeth, the traditional slot skewing and the more recent slot opening skewing, magnet segmentation, optimizing the magnet pole arc width and others). The main disadvantage of this approach is that motors have to be rebuilt, which in turn will generate higher costs and will require longer production/research time. Moreover, rotor skewing requires PM with particular shapes, which are not easy to be manufactured, very expensive, and difficult to be magnetized. The second category involves specific techniques for minimizing the pulsating torque (such as harmonic injection or back-EMF inversion) which are highly dependent on the accuracy of the sensors used to measure the periodic disturbances wave patterns. However, the second type of minimization methods offers lower hardware costs and lesser implementation times.

This paper proposes a controller-based pulsating torque minimization technique. In order to minimize the effects of periodical disturbances, a solution that uses control signal reshaping is chosen. The method is based on current disturbance observation and compensation of particular harmonics. Cogging torque, ripple torque and mechanical beating are first detected and described using a Fourier series expansion. An extended off-line analysis of the collected data is made in frequency domain, based on frequency spectra of the motor current output, at different levels of rotational frequencies. This analysis emphasizes the periodic disturbances, which depend greatly on the rotor position, as opposed to the operating angular velocity. After the off-line analysis, we propose an online system for minimizing the cogging torque.

**Fig 1:**
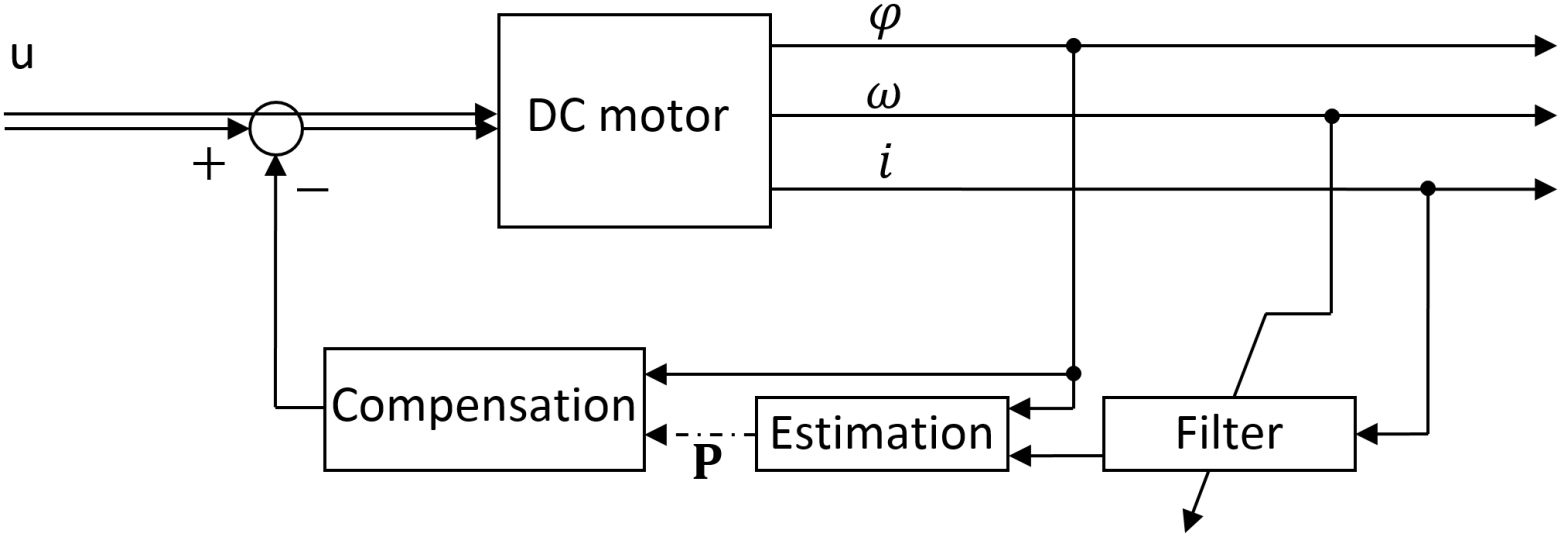
Block diagram of compensation loop.

Several studies focused on online estimation methods. I.e. in [4], researches successfully modeled the dynamic voltage behavior of a lithium-ion battery, based on the recursive least square algorithm. The state of charge of a lithium-ion battery was also estimated online using Kalman filter and its variations in [5, 6, 7]. In our case, the compensation requires online parameter identification of the periodic disturbances model, as the disturbing harmonics are sensitive to initial conditions of the rotary motion. For this approach, an adaptive filter based on look-up tables and a recursive estimation algorithm are designed and implemented. The filter is able to eliminate the detected harmonics from the motor current, which contributes to the reduction of developed torque pulsation, for any motor velocity. The recursive estimation algorithm implemented based on the normalized gradient identifies the model parameters. The compensating signal is directly added to the servo motor control signal. The adaptive filter provides the particular disturbing harmonic in the measured motor current during the estimation phase as a function of base rotational frequency. The recursive estimation algorithm implements the normalized gradient method. The estimated disturbance model reshapes the control signal to suppress the disturbing harmonics. The control loop is shown in Fig 1, where *u* is the control voltage, *φ* is the angular position, *ω* is the rotational velocity, *i* is the motor current and *P* is the parameters set.

## Literature Overview

### 2.1. Preliminary analysis of the periodic disturbances

PM motors have various advantages as opposed to other motor types (separately excited motors, compounded motors), such as robustness, reliability, high power factor and efficiency, good power/volume ratio and cheap maintenance costs [8]. They do however suffer from effects of pulsating torque, which is defined in literature as the sum of different perturbations [2]:

- The cogging torque—generated by the interaction between the PM of the rotor and the stator slots. It results from the varying air gap reluctance due to the slot positioning. The rotor has the tendency to align in a number of stable positions, even when the motor is not excited [9]. Also known as detent or “no-current” torque, it is an undesirable component. Cogging is higher at low speeds. This imperfection is greatly dependent on the motor design [10].
- The torque ripple—caused by the non-sinusoidal shapes of the current in DC motors, the slight mismatch in shape between the back-EMF shape and the current shape, the presence of the stator slots and the irregular magnetic field of the permanent magnets.
  In order to keep a constant torque on the rotor, the current through the coil needs to be reversed every half turn. In DC motors, multiple coils are present and the resultant torque is always greater than zero, but it is not constant. This angle dependent variation is called torque ripple [11].
- Mechanical beating—caused by eccentricities of the motor construction and due to the motor bearings used for axial rotation of the rotor shaft. Beating is usually detected as being the first harmonic in the frequency spectrum and is higher when motor operates at low velocities. Beating is a vibration phenomenon which occurs when 2 harmonics motions of the same amplitude, but slightly different frequencies, are applied to a mechanical system [12–14].

$$x_{1}=Xcos\omega t$$ (1)

$$x_{2}=Xcos\left(\omega t+\Delta \omega \right)$$ (2)

The resultant motion of the mechanical system will be the superposition of the input vibration x_1_, x_2_ which is:

$$x=2Xcos\left(\frac{\Delta \omega }{2}\right)tcos\left(\omega +\frac{\Delta \omega }{2}\right)t$$ (3)

This vibration causes the mechanical beating effect. The frequency and period of the beat is:

$$f=\;\frac{\Delta \omega }{2\pi }cycles/sec$$ (4)

$$T=\;\frac{2\pi }{\Delta \omega }sec$$ (5)

The main advantages and disadvantages of some of the most important design minimization techniques of pulsating torque are reviewed in Section 2.2. Section 2.3 is reviewing various control-based methods for pulsating torque minimization reported by the scientific community in recent years. We emphasize on the advantages and disadvantages of the approaches enclosed within the proposed algorithm.

### 2.2. Motor design techniques for pulsating torque minimization

The first approach for pulsating torque minimization presumes adjusting the PM motor design. The main idea behind this set of techniques is to build the motor in such a way that it directly addresses the sources of the perturbations [15].

One of the most popular minimization techniques is skewing. Skewing can be applied to the stator slots or alternatively, to the permanent magnets [2, 16, 17]. Skewing reduces the variation of reluctance seen by the rotor magnets and hence, the cogging torque effect. Skewing effectiveness goes up to 99% (due to the end effect, the cogging torque cannot be canceled completely by skewing [18]). It improves the stator windings distribution and reduces higher order back-EMF harmonics [19]. One of the main drawbacks of this method is that it requires PM with particular shapes which are not easy to be manufactured. As all other minimization techniques, it also produces a torque loss. It is also dependent on the number of slots per pole—if PM motors have just a few slots, skewing produces an important torque loss.

Pole shifting is another classic method that is used to reduce the cogging torque. The magnet poles are displaced along the rotor circumference in order to compensate each other. Magnet consumption and total costs are reduced. As disadvantages, this technique causes larger torque ripple effects, as well as a larger generator inductance [20].

Magnet segmentation is a minimization technique in which the distribution density of the air-gap flux is customized by segmenting the magnet poles into several elementary magnet blocks. By choosing either the appropriate elementary magnet block span or the relative position of the magnet blocks, the cogging torque may be significantly reduced. As major drawback, magnetic segmentation deteriorates the air gap flux density and decreases the output torque [18, 21].

Another cogging torque minimization approach is based on the optimization of the pole arc coefficient. Using a combination between domain elimination algorithm and finite-element method, this approach explores an entire feasible region domain in a systematic way, searching for a global minimum [22]. The reported results however still need refinement, as the study suggests the need to rebuild the shape of the stator.

Cogging torque has also been reduced using less common techniques such as stator teeth and stator slot pairing [23, 24] or teeth notching [25].

A solution that leaves cogging torque aside and aims to reduce torque ripple for once is rotor shape modification, more exactly, flux barrier modification, yet this may only be applied to interior permanent magnet motors [26].

As one call infer, despite the wide range of motor design techniques that are available for reducing ripple torque components, there are many cases when they are either not sufficient or appropriate to achieve the required minimization of pulsating torque. Most of the times, there is a design tradeoff among magnetic loading, electrical loading, and financial costs. Control-based techniques for pulsating torque minimization try to deal with this issue.

### 2.3. Control-based techniques for pulsating torque minimization

There are several classic control techniques that deal with pulsating torque minimization. All of them focus on altering input parameters for producing a smoother torque.

One of the first control algorithms is based on programmed excitation waveforms [27, 28]. The main idea is to produce waveforms that neutralize the summation of each harmonic. We refer to harmonics as to the periodic waves forming the signal outputted by the motor. But for sinusoidal PM motors, this method is not suitable since phase currents need to be individually controlled by the sinusoidal drive, as opposed to Brushless DC (BLDC) Motors.

An algorithm fairly similar to programmed excitation waveforms is harmonic injection [29]. However, this approach complicates the design of the motor modules, and targets only specific ripple components [30], having a weak performance in practical applications.

Torque ripple minimization was also targeted in [31], where a technique which is based on PWM excitation pulses produced during the time when each motor phase would normally be unexcited is introduced. The technique suffers due to the permanent customization which needs to be executed for each set of motor parameters.

Another classic method for reducing torque ripple is based on non-ideal back electromotive force (EMF) waveforms. It has been shown that torque ripple can be minimized in the case of BLDC motors [32–34]. Using the back-EMF technique, there is no need to analyze any harmonic of the flux or back-EMF waveforms themselves, but as all other techniques described above, it poses substantial challenges in real practical situations, due to its open-loop nature.

Other control techniques which work great in specific cases include direct torque control [35] and closed-loop speed regulation [36].

One of the most diversified classes of techniques (which also includes the method proposed in this paper) is the estimator/observer one. Basically, the control of input parameters is performed considering a measured feedback. Several studies have used this approach [37, 38], with drawbacks such as weak performance at low speeds, required pre-knowledge of motor’s design and functional parameters or temperature control [39].

## Overview of the Proposed System

### 3.1. Hardware and software prerequisites for theoretical and experimental studies

The main platform used in the experiments presented in this paper is composed of 5 modules (also used in similar studies [35]):

- A data acquisition board with a real time framework
- An amplifier
- A DC servo motor
- An encoder
- A shunt resistance

Implementation was conducted in MATLAB (Real Time Workshop) and only standard elements and embedded functions of Simulink were used for this study.

#### Experimental test bed

The data acquisition board installed on the host computer has a sample rate of 4 kHz. The target is a DC servo motor with a shrunk-on disk rotor, eight poles, 4 brushes and with the rated power output of 80W and rated speed of 3000 rpm. The motor current is measured by the voltage drop across a shunt resistance. The motor shaft position and direction of the rotation are provided by a digital single turn rotary encoder with 13 bit resolution. The angular velocity results from time derivation of the shaft position [40].

**Fig 2:**
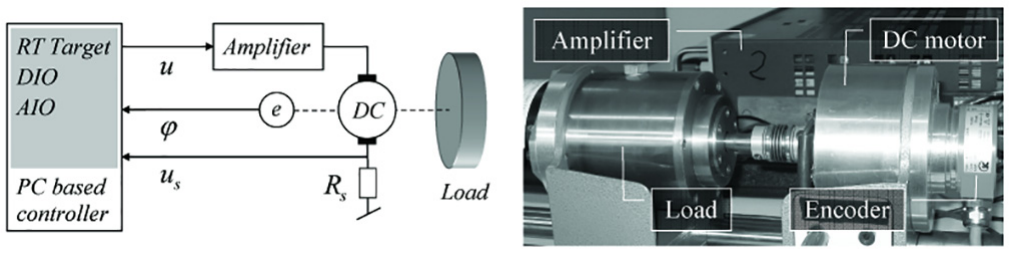
System overview and experimental test bed.

The encoder is firmly connected to the back-shaft of the motor, so that both the shaft and the encoder disk rotate at the same angular velocity (Fig 2).

#### DC motor

As in most PM motors, the rotor windings are welded onto a thin disc. The disc rotates in the air gap between pairs of PMs arranged at disk’s extremities. A radial set of fans follows the winding displacement. The magnets are alternatively arranged in order to compensate the magnetic fields in each air gap. Two end caps made of iron close the magnetic circuit and hold together the magnets. Current coming from the motor brushes passes through commutator segments printed on the disc and reaches the rotor windings. As opposed to traditional electric motors which have a radial magnetic flux and an axial current flow, in our case, the magnetic field is axial and the current flows radially from the center axis to the edge of the disc and back. The tangential force which actions the disc is created because of the interaction of the wires which carry the outgoing and the return currents with the adjacent magnets.

### 3.2. Modelling the system

**Fig 3:**
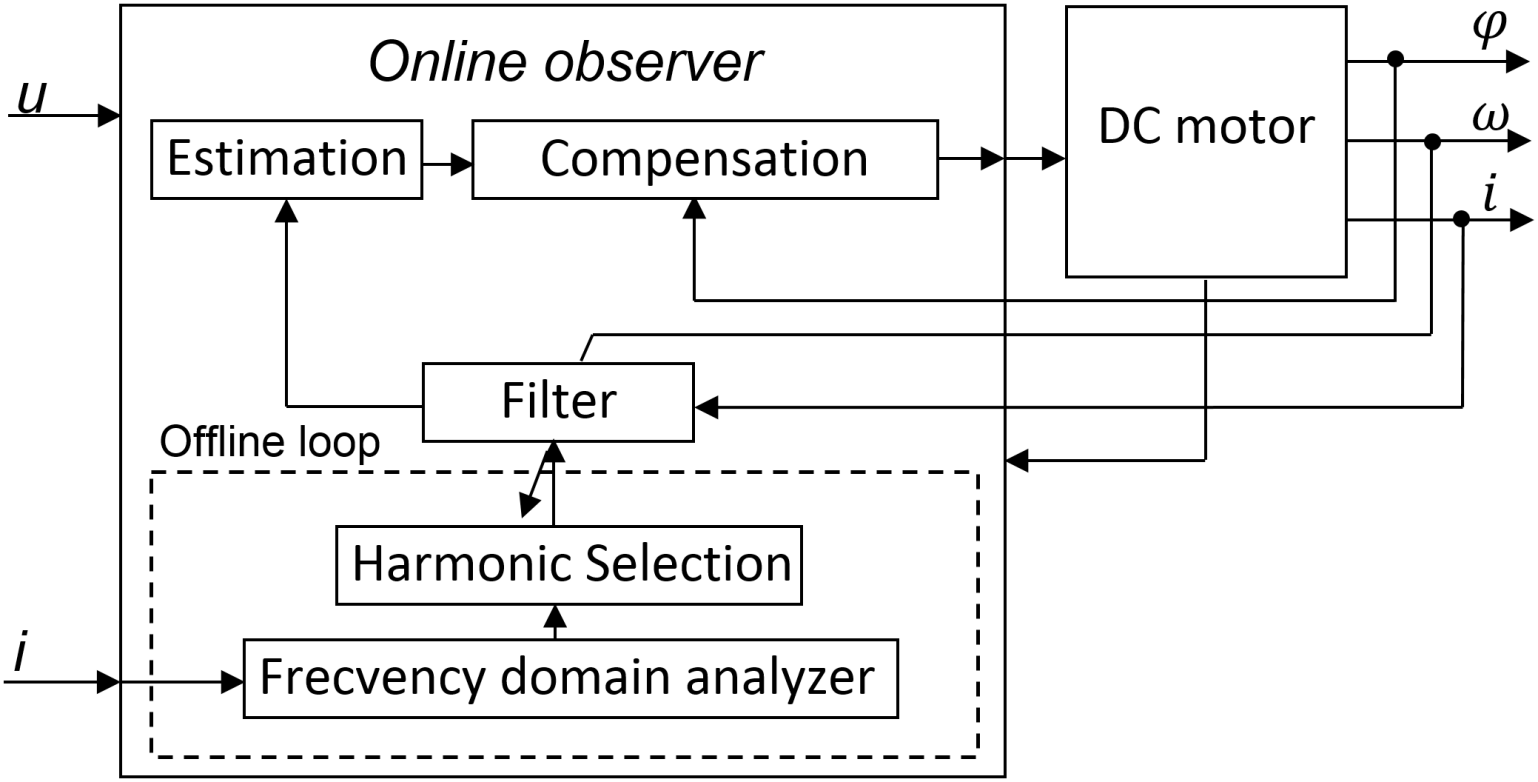
System architecture.

The proposed algorithm presented in Fig 3 expands the diagram from Fig 1. The “Online Observer” block acts as a buffer between the input and the DC motor. First, all harmonics are analyzed offline, based on the output given by the “Frequency domain analyzer” block, after a fast Fourier computation, residues removal and normalization. The most significant ones are selected in “Harmonic Selection” block, and offered for online filtering, process that happens within the “Filter” block. The system runs through a continuous optimization loop created between the, “Filter”, “Estimation”, “Compensation” and “DC motor” blocks. The compensating harmonics that are meant to attenuate the most significant harmonics are estimated in “Estimation” block and sent to “Compensation” block. Thus, the proposed algorithm has certain similarities with harmonic injection and observer/estimation techniques. The process happens continuously, as the output from the DC motor is sent back to the “Online Observer”. The pulsating torque minimization process is thus separated in 2 distinct phases: the offline loop which runs for a while before calculating the required data, and the online compensation which starts after the calculation is completed.

#### DC motor model

In order to design the DC motor model, dynamic equations are included. This mathematical model is derived considering the electrical and the mechanical parts of the system. The two characteristic equations are:

$$u\left(t\right)=L\frac{di}{dt}+R_{m}i\left(t\right)+K_{e}\omega \left(t\right)$$ (6)

$$K_{m}i\left(t\right)=J\frac{d\omega }{dt}+K_{d}\omega \left(t\right)+\tau_{l}+\tau_{f}$$ (7)

Eq (6) represents the equivalent motor electrical circuit which can be derived by using Kirchhoff’s voltage law around the electrical loop (the sum of all voltages around a loop must equal zero). According to Ohm’s law, the voltage across the resistor can be represented as *R*_*m*_*i*(*t*) where *i*(*t*) is the armature current. The voltage across the inductor is proportional to the change of current through the coil with respect to time and can be written as $L\frac{di}{dt}$, where *L* is the inductance of the armature coil. Finally, the back electro-motive force (EMF) can be written as *K*_*e*_*ω*(*t*) where *ω* is the rotational velocity of the armature.

Eq 7 performs an energy balance, as the sum of the torques of the motor must equal zero. The electromagnetic torque is assumed proportional to the current through the armature winding and can be written as *K*_*m*_*i*(*t*) where *K*_*m*_ is the torque constant, which depends on the flux density of the fixed magnets, the reluctance of the iron core, and the number of turns in the armature winding. $J\frac{d\omega }{dt}$ is the torque due to rotational acceleration of the rotor, where *J* is the inertia of the rotor. The torque associated with the velocity is written as *K*_*d*_*ω*(*t*) where *K*_*d*_ is the damping coefficient associated with the mechanical rotational system of the machine. The system load and friction is are denoted by *τ*_*l*_ and *τ*_*f*_.

The overall model has six independent parameters, of which the inductance L = 25*10^*−6*^ *(H)* is obtained from the manufacturer data sheet, and the remaining five parameters are identified experimentally.

#### Periodical disturbances model

For describing the periodic disturbances, a simple cogging torque model is introduced. As presented, cogging torque appears due to the slotting displacement. From modeling point of view, the cogging torque is caused by the variation of the magnetic energy of the field due to the interaction of PMs with the mechanical angular position *φ* of the rotor [1]. For simplicity, it can be considered that cogging torque is a sum of interactions of each edge of the motor’s PMs with the slot openings.

Because the approach proposed for compensation is based on reshaping the control signal, an analytical model of periodical disturbances is described using Eq 8:

$$\tau_{cog}\left(\varphi \right)=\sum_{i=1}^{N}A_{i}\text{sin}\left(i\varphi +\sigma_{i}\right)$$ (8)

where *A*_*i*_ and *σ*_*i*_ are the parameters of the model.

### 3.3. Measurements

For the analysis of periodic disturbances, first, a set of data is collected from DC motor with the help of laboratory equipment. The experimental measurements are composed of 4 types of measured signals:

- Control signal in (V)
- Angular position in (deg)
- Angular velocity in (rad/s)
- Motor current in (A)

The minimum voltage of the DC motor when the stick slip effect is not present is 4V. The maximum voltage is 14V, obtained from the manufacturer’s data sheet. Thus, the measurements are taken in the interval 4-14V with a step of 0.5V, resulting a set of 21 measurements. All the measured signals are discrete-time signals sampled with a 4kHz frequency.

**Fig 4:**
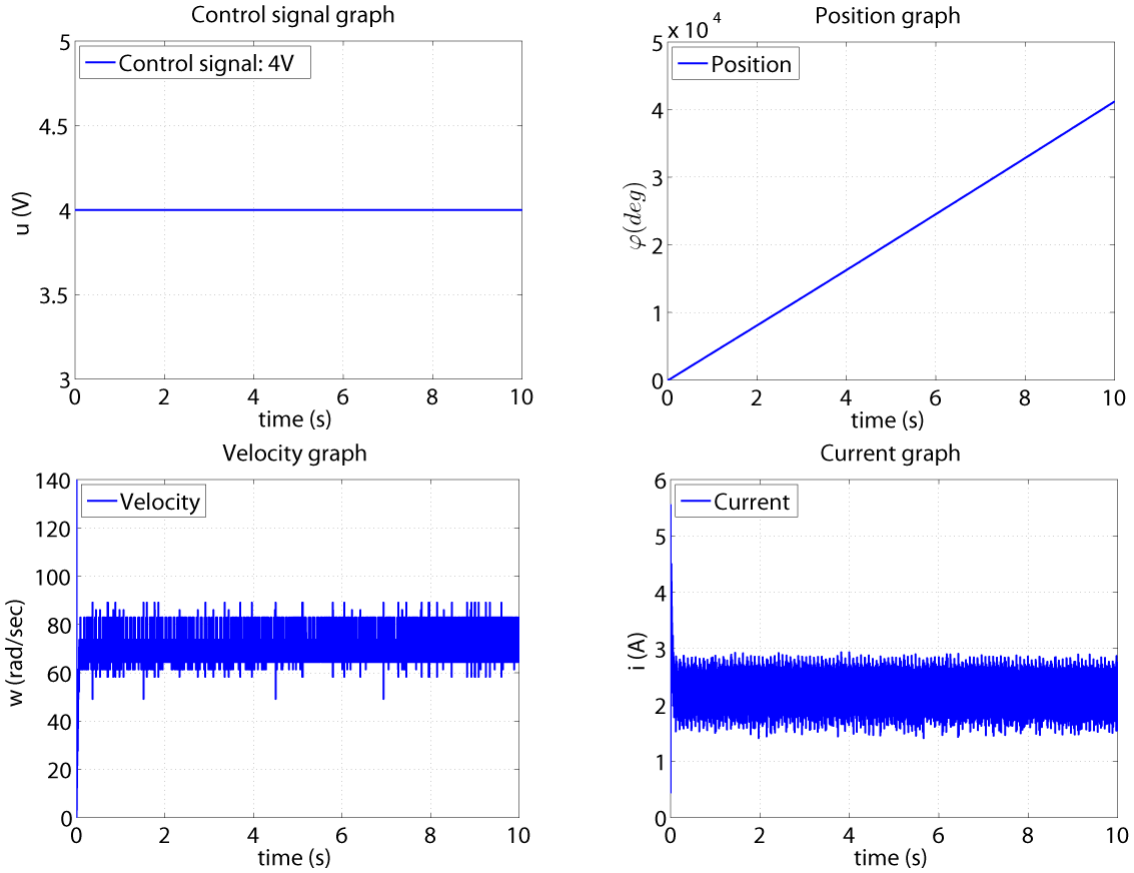
Collected measurements for u = 4V.

As example, in Fig 4 are illustrated 4 graphics of the measured signals for an input of 4V. All collected measurements are stored in MATLAB workspace as a time structure.

## Frequency Domain Analysis

### 4.1. Fast Fourier transform

In order to analyze the current behavior, the signal is transformed in frequency domain, a necessary step for the extraction of useful information. The frequency domain is based on the concept of Fourier series, representing sinusoidal components with different frequencies, which provides insight into data patterns. The amplitude and phase of each sinusoidal component in the sum determines the relative contribution of that frequency component to the entire signal. To compute the frequency spectrum graph of the current signal, the Fast Fourier Transform algorithm is used (an enhanced version of Discrete Fourier Transform).

**Fig 5:**
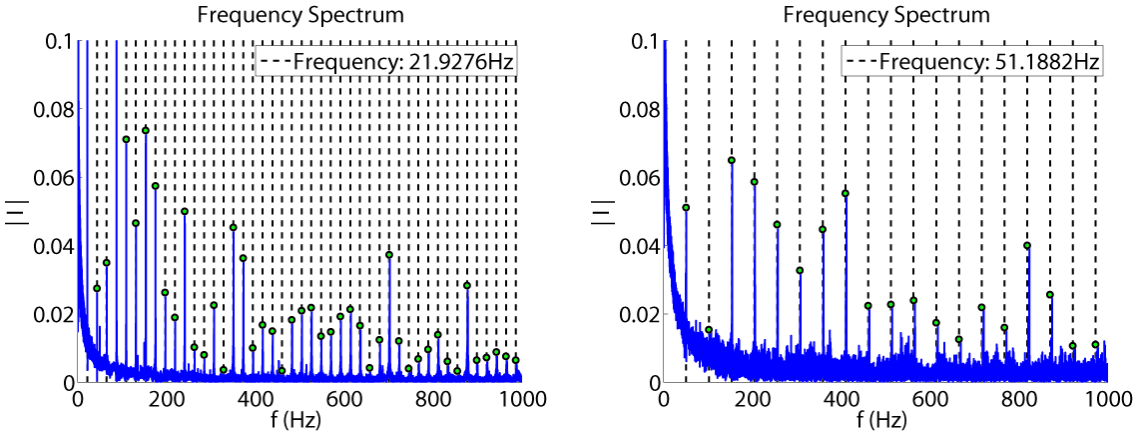
Frequency spectrum for u = 6V (a) and u = 14V (b).

After completing this phase, one can now distinguish among frequency components of the current signal buried in a noisy time domain. In Fig 5, the current is represented in frequency domain for different values of the input signal. X axes are representing the frequency; Y axes are representing the current. The peaks which can be seen in each graph may have as source periodic disturbances, but also other components like i.e. the white noise from the public electric power line.

### 4.2. Harmonic computation

In real life situations, there are a lot of processes which can be modeled by oscillating behaviors. Such is the case of a rotary machine. Some of the occurring signals are periodic or at least contain periodic parts. A harmonic steady state oscillation can be described by a sine function shifted in phase [41]:

$$y\left(t\right)=\;y_{0}\text{sin}\left(2\pi f_{0}t+\varphi \right)=y_{0}\text{sin}\left(\omega_{0}t+\varphi \right)$$ (9)

with amplitude *y*_0_, frequency *f*_0_ = 1/*T*_*p*_ (with *T*_*p*_ the duration of one cycle of periodic signals), angular frequency *ω*_0_ = 2*πf*_0_ and phase angle *φ*.

When damping the harmonic oscillation:

$$y\left(t\right)=\;y_{0}e^{-\delta t}\text{sin}\left(\omega_{0}t+\varphi \right)$$ (10)

with *δ* the damping constant. The simplest way of describing combined oscillations is by superposition [42]:

$$y\left(t\right)=\sum_{v=1}^{n}y_{0}e^{-\delta_{v}t}\text{sin}\left(\omega_{v}t+\varphi_{v}\right)$$ (11)

where *v* is the harmonic number.

The harmonics that need to be compensated are the ones which have as source the periodic disturbances described in Section 2.1.

To identify which harmonics are these and to determine the components of the signal, a grid is applied over the frequency spectrum. The grid and the selected maximum value for each harmonic are illustrated in Fig 5. The spacing between the grid lines is equal with the value of the rotation frequency of the motor. As stated above, all measured discrete-time signals are sampled with 4kHz sampling frequency. As Nyquist frequency is half the sampling frequency of a discrete time signal, the resulted frequency spectrum is 2000Hz. At an input of 4V, the motor will rotate with a measured frequency of 11.6Hz, and the grid will have 172 harmonics (2000/11.6). At an input of 14V, with a frequency rotation will be 64Hz, and the grid will have 31 harmonics.

The developed search algorithm selects the highest peak in a range of 20 percent left and right from each grid line. Selected values represent the harmonics magnitudes and are stored in a matrix for further analyzing within MATLAB environment. The obtained matrix has the form presented in Eq 12.

$$M_{i,j}=\left(\begin{array}{cccccccc} i,j & 1 & 2 & \cdots & 31 & \cdots & 171 & 172\\ 1 & value & value & \cdots & value & \cdots & value & value\\ 2 & value & value & \cdots & value & \cdots & value & 0\\ \vdots & \vdots & \vdots & \cdots & \vdots & \cdots & \vdots & \vdots \\ 21 & value & value & \cdots & value & \cdots & 0 & 0 \end{array}\right)$$ (12)

in which the number of rows *i* represents the set of measurements (21 measurements, as explained in section 3.3) and the columns *j*, the number of harmonics (minimum 31, maximum 172, depending on the voltage). The algorithm searches for the highest peaks and returns a different number of harmonics *n*, depending on the measurement frequency. Following, we make the assumption that by minimizing only the commune (strongest harmonics) components, most residuals will also be removed. The resulted matrix contains 31 magnitude values of the strongest harmonics, since 31 is the minimum number of harmonics identified at any given voltage.

$$M_{i,j}=\left(\begin{array}{ccccc} i,j & 1 & 2 & \cdots & 31\\ 1 & value & value & \cdots & value\\ 2 & value & value & \cdots & value\\ \vdots & \vdots & \vdots & \cdots & \vdots \\ 21 & value & value & \cdots & value \end{array}\right)$$ (13)

The obtained harmonics need to be compared between themselves, thus the values need to be normalized. The normalization is performed using the maximal value for each corresponding measurement *i*:

$$M_{i,j}=\left(\begin{array}{ccccc} i,j & 1 & 2 & \cdots & 31\\ 1 & value/max_{1} & value/max_{1} & \cdots & value/max_{1}\\ 2 & value/max_{2} & value/max_{2} & \cdots & value/max_{2}\\ \vdots & \vdots & \vdots & \cdots & \vdots \\ 21 & value/max_{21} & value/max_{21} & \cdots & value/max_{21} \end{array}\right)$$ (14)

Now that each magnitude value is scaled from 0 to 1, it can be seen how these are distributed for different velocities. For each harmonic, a set of 21 values can be analyzed, corresponding to the set of measurements.

### 4.3. Harmonic distribution

To see how the harmonic magnitude values are distributed for different input signals, the probability density function for the normal distribution described in equation Eq 15 is used. The Gaussian function is defined by two parameters, the mean (*μ*) and variance or standard deviation squared (*σ*^*2*^).

$$\varphi_{\mu ,\sigma^{2}}\left(x\right)=\frac{1}{\sigma \sqrt{2\pi }}exp^{-\frac{{\left(x-\mu \right)}^{2}}{2\sigma^{2}}}$$ (15)

The Gaussian function representation is a symmetric bell shape curve that quickly falls to plus/minus infinity. In this analysis, magnitude values’ dispersion is relevant for establishing which harmonics are dependent on velocity. High dispersion represents a velocity dependent harmonic; small dispersion represents a non-dependent harmonic. The mean *μ* represents how strong the peaks of a considered harmonic are over all 21 measurements.

**Fig 6:**
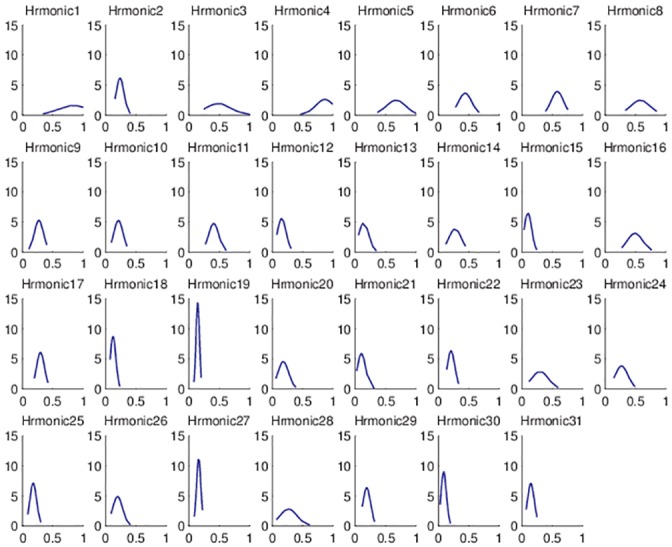
Normalized distribution of harmonics.

The most significant harmonics are the ones which have the average value ≥ 0.5. For the specific case of the motor used in the experiments conducted within this study, the harmonics selected for compensation are 1, 3, 4, 5, 7, 8 and 16. These selected values were expected to be found, based on motor’s design parameters. The first harmonic represents the output of the mechanical beating phenomenon; the 4^th^ harmonic has as a source the 4 brushes of the motor; the 8^th^ harmonic is dominant because of the 8 magnetic poles. Taking into consideration the principle of superposition and amplitude modulation, harmonics 3, 5 and 7 can be consequently be explained. Thus, in Fig 6 which presents the distribution of the normalized harmonic values, it can be seen that the selected harmonics pass well over the 0.5 limit and are suitable to be minimized.

## Adaptive Filter Design

### 5.1. Filter design

When building a digital signal filter, one needs to remove unwanted parts such as noise, or to extract useful components such as specific harmonics. When designing an adaptive filter, its parameters need to adapt to certain characteristics of the signal such as velocity, using i.e. look up tables.

In our case, a band-pass filter can be used to filter particular harmonics. This type of filter passes frequencies within a certain range and attenuates frequencies outside that range. This filter can be used to pass only frequencies that belong to the selected harmonics.

An ideal band-pass filter has a flat pass band, completely attenuates all frequencies and has an instantaneous transition. In real situations, the band-pass filters don’t completely reject all frequencies outside the desired range. There is a region just outside of the pass band where frequencies are not rejected, known as the filter roll-off [43]. In our case, the filter roll-off has to be as narrow as possible, for obtaining as little white noise as possible in the filtered output. Nonetheless, filtering has to be achieved with minimum filter order for as little delay as possible, especially in online scenarios.

Both types of digital filters (Finite Impulse Response (FIR) and Infinite Impulse Response (IIR)) are taken into consideration for implementing the filter block.

#### FIR filter

One of the main advantages of FIR filters is that they have the same delay for any filtering frequency, as they have a linear phase. While these filters are very stable, their filtering performance varies with the filter order. FIR filter order is directly related to the number of previous inputs which need to be stored in order to calculate the current output:

$$y\left(n\right)=\;b_{0}x\left(n\right)+b_{1}x\left(n-1\right)+b_{2}x\left(n-2\right)+\ldots +b_{N}x\left(n-N\right)$$ (16)

where *N* is the filter order and *b*_*n*_ are the filter coefficients. These coefficients are the ones that determine the characteristics of a FIR filter.

**Fig 7:**
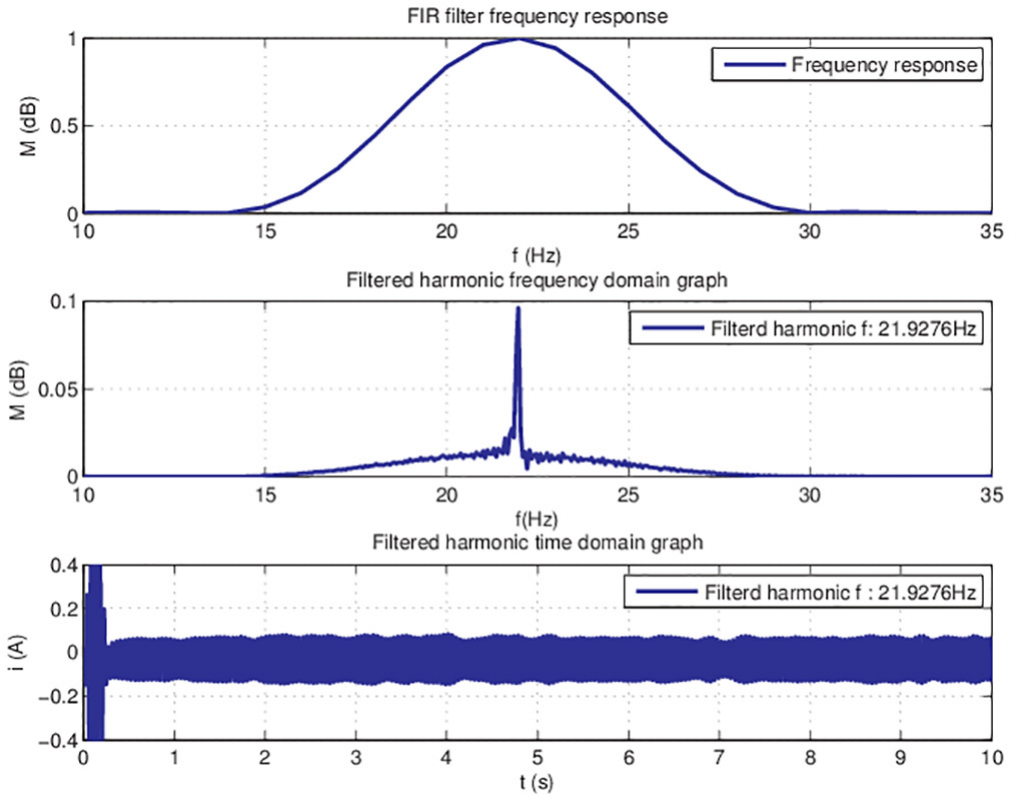
FIR filter frequency response and filtered data.

For this particular case, the filter was tested off line. Filter coefficients were calculated with *b* = *fir*1(*n*, *W*_*n*_) MATLAB function, where n is the order of the filter and *W*_*n*_ = [*w*1 *w*2] is a two-element vector representing the cutoff frequency. The function returns the coefficients for a band-pass filter with the pass band *w*1 < *w* < *w*2. After the filter design process has generated the filter coefficient vector *b*, the result is tested for data response (Fig 7).

The implementation process outlined that a 500 filter order is needed for good quality filtering. The required filter order is too big and the corresponding delays are affecting the online operation. Therefore, the possibility of using an IIR filter is investigated, with the aim of obtaining an equivalent filtering quality at a lower filter order.

#### IIR filter

In the case of IIR filters, using feedback, a set of design specifications can be met with a far smaller filter order (vs. comparable FIR filters). IIR filters typically offer less delay elements than other implementations. With IIR filters, faster computation comes with certain drawbacks: IIR filters cannot have a perfectly linear phase. Phase linearity is important in this case, where the time-domain filtered waveform is used for estimation purposes.

IIR is a recursive filter which in addition to input values, also uses previous output values. These are stored in the processor’s memory, and used as in Eq 17:

$$\begin{array}{l} y\left(n\right)=\;b_{0}x\left(n\right)+b_{1}x\left(n-1\right)+b_{2}x\left(n-2\right)+\ldots +b_{N}x\left(n-N\right)\\ -a_{1}y\left(n-1\right)-a_{2}y\left(n-2\right)-\ldots -a_{M}y\left(n-M\right) \end{array}$$ (17)

where *N* is the feed forward filter order and *M* is the feedback filter order.

**Fig 8:**
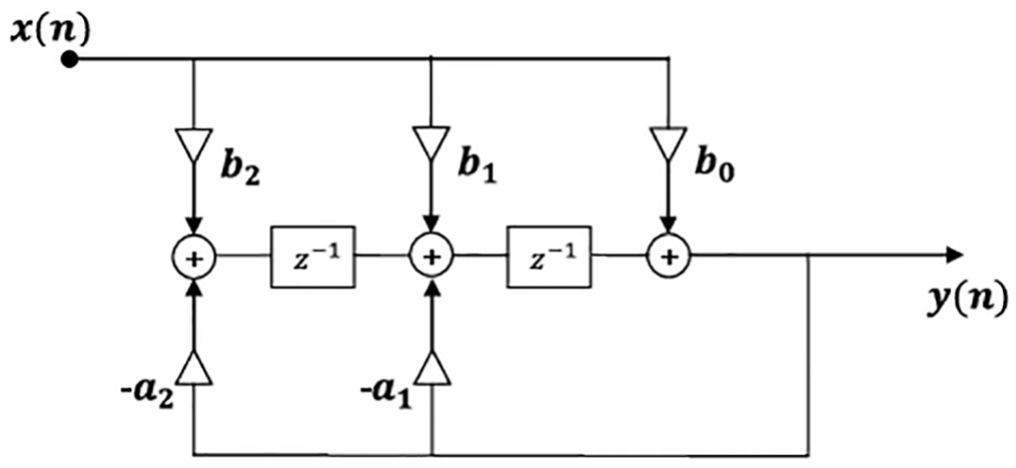
Second order IIR filter.

The design technique used to implement the IIR filter is based on Butterworth’s work [44]. This approach concludes in a maximally flat IIR filter. For this reason, the only design parameters are the cutoff frequency and the filter order. For designing a band-pass filter with pass band *w*1 < *w* < *w*2, minimum order is 2. The filter makes use of two previous inputs *x*(*n*−1) and *b*_2_*x*(*n*−2) and two previous outputs *y*(*n*−1) and *y*(*n*−2). The filter structure is presented in Fig 8.

**Fig 9:**
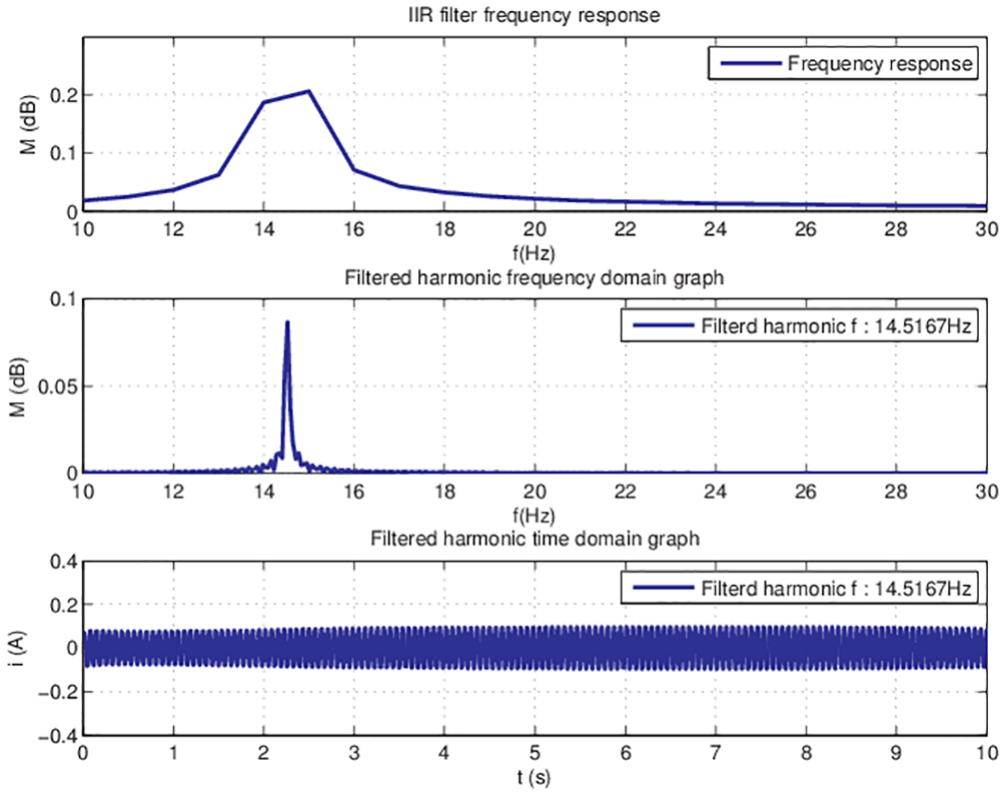
IIR filter frequency response and filtered data.

This implementation is tested off-line for filtering one harmonic. The coefficients of the filter are calculated with [*b*, *a*] = *butter*(*n*, *W*_*n*_) MATLAB function, where *n* is the filter order and *W*_*n*_ = [*w*1 *w*2] is a two-element vector that represents the cutoff frequencies. The frequency response for filtering the first harmonic is represented in Fig 9.

The results obtained with this particular filter design are showing that the quality of the filtered signal is acceptable. This implementation is chosen and implemented in Simulink.

### 5.2. Filter implementation

Within our system architecture, the “Filter block” must be able to perform the filtering of selected harmonics on-line, for any angular velocity. Thus, it needs to be able to adapt its coefficients based on the instantaneous angular velocity input. This can be done by means of lookup tables. A lookup table block uses an array of data to build a function approximation between input and output values [45]. The harmonics that need to be filtered are in the range of 0 to 1000 Hz. For this specific range, filter coefficients are calculated off-line and stored in lookup tables. For a given input value (in this case, the velocity), a”lookup” operation is performed, in order to retrieve the corresponding output values from the table which are the coefficients for the filter. If the lookup table does not contain the input values, the block estimates the output based on nearby values, by linear interpolation. By using the lookup tables method, the filter becomes adaptive, changing its coefficients based on the velocity input signal. For any motor velocity, the most significant harmonics can be filtered out from the original current signal.

**Fig 10:**
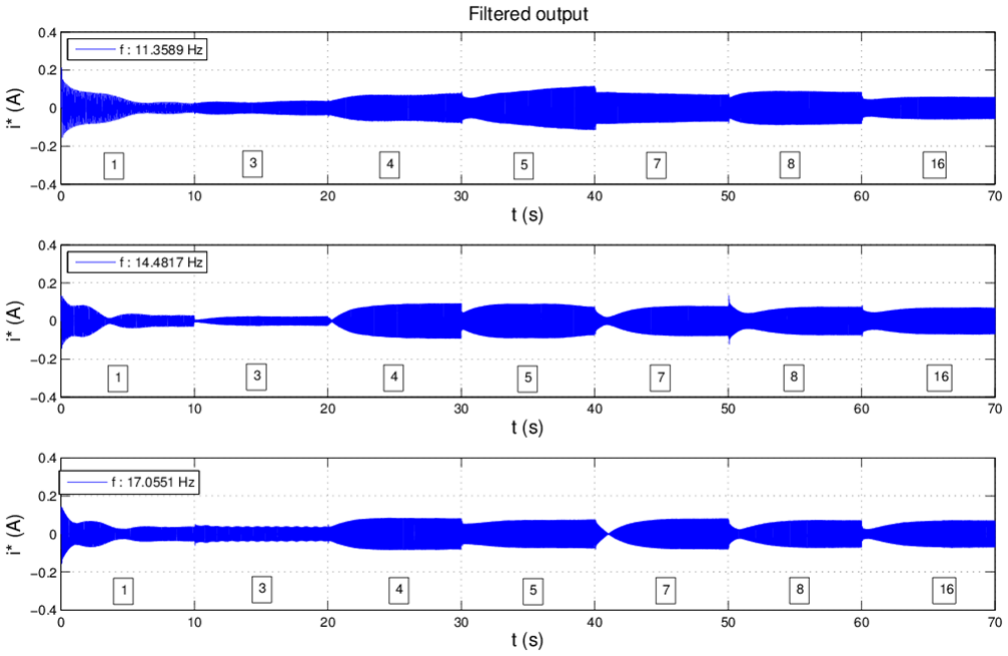
Filter output for u = 4V, 4.5V and 5V.

In Fig 10 filter output is represented for different velocities. For each harmonic, filtering is done for 10 seconds. The only information about the filtered signal is the frequency *f* of the sinusoid. Filtering is not ideal, thus a small amount of white noise and oscillations are present into the filtered signal, resulting in a noisy sinusoid with a known frequency. To create the compensation signal, information about the amplitude and phase for each filtered harmonic is needed. The next section presents the proposed approach and implementation for on-line identification of these parameters.

## Recursive Estimation Algorithm

### 6.1. General form

**Fig 11:**
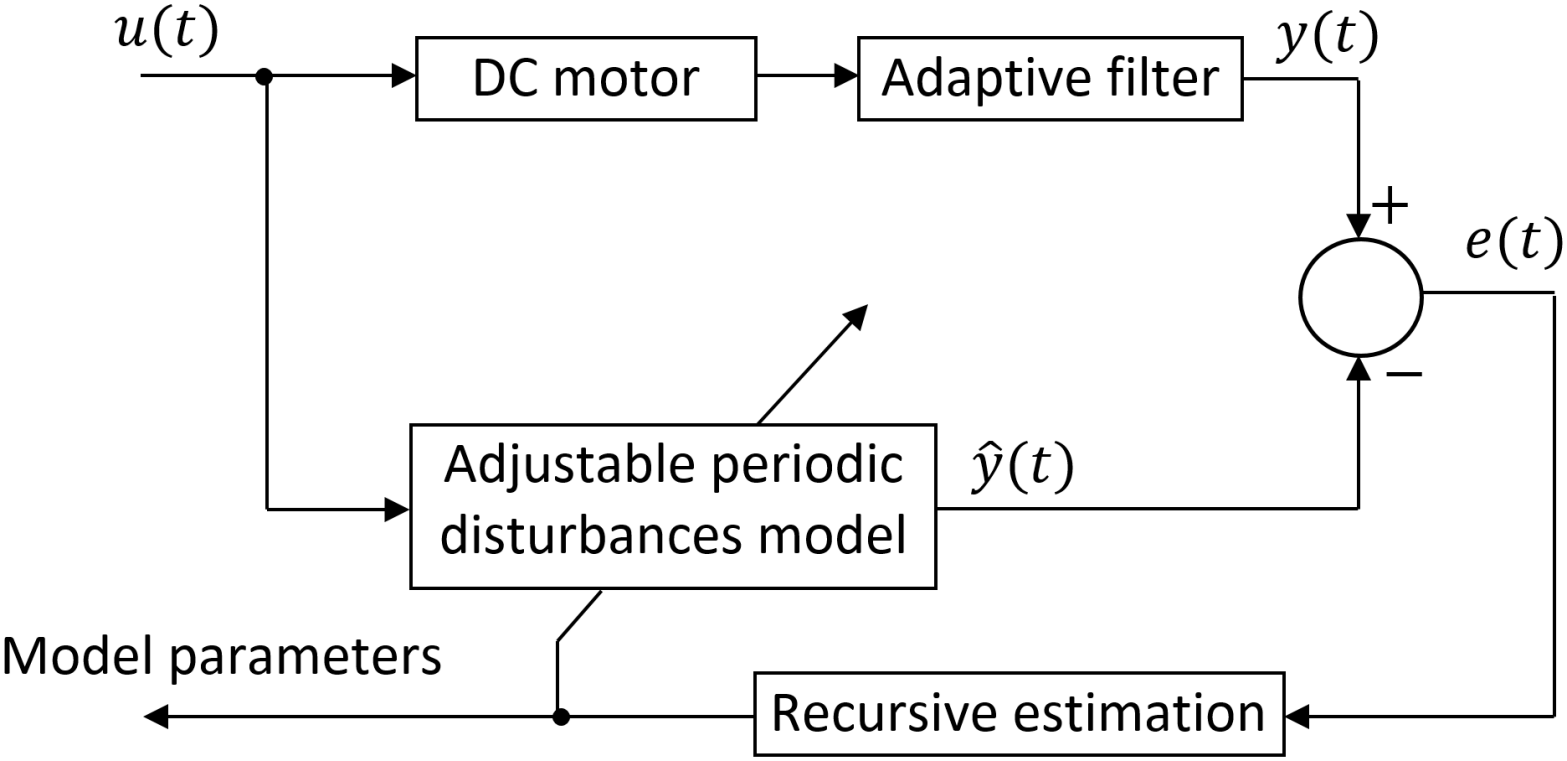
Principle of model parameters estimation.

The block diagram of the principle behind the implementation of parameter estimation module is presented in Fig 11. The difference between the periodical disturbances model output $\hat{y}(t)$ and the filtered signal output *y*(*t*) represents the observation error *e*(*t*). Eq 18 represents the general form of a recursive estimation algorithm [46]:

$$\hat{\Theta }\left(t\right)=\;\hat{\Theta }\left(t-1\right)+K\left(t\right)\left(y\left(t\right)-\hat{y}\left(t\right)\right)$$ (18)

where $\hat{\Theta }(t)$ is a vector of two values, amplitude and phase, *y*(*t*) is the filter output, $\hat{y}(t)$ is the prediction of *y*(*t*) based on observations up to time (t-1), and *K*(*t*) is:

K(t)=Q(t)ψ(t) (19)

*K*(*t*) determines how much the current observation error $y(t)-\hat{y}(t)$ affects the update of the parameter estimate. The estimation algorithm tries to minimize the observation error.

The normalized gradient *Q*(*t*) is the product of the gain *λ* and the identity matrix, normalized by the magnitude of the gradient *ψ*(*t*). After choosing *Q*(*t*), the parameters are updated in the negative gradient direction, where the gradient is computed as in Eq 20:

$$Q\left(t\right)=\frac{\lambda }{{\left|\psi \left(t\right)\right|}^{2}}I$$ (20)

where *I* is the 2x2 identity matrix.

### 6.2. Gradient vector

The periodic disturbances of *i* number of harmonics are computed by a function of two variables—amplitude *A*_*i*_ and phase *σ*_*i*_:

$$F\left(A_{i},\sigma_{i}\right)=A_{i}\text{sin}\left(i\varphi +\sigma_{i}\right)$$ (21)

To compute the gradient vector of a function with two parameters, the partial derivations are considered:

$$\psi =\left(\begin{array}{c} \frac{\vartheta F\left(A_{i},\sigma_{i}\right)}{\vartheta F\left(A_{i}\right)}\\ \frac{\vartheta F\left(A_{i},\sigma_{i}\right)}{\vartheta F\left(\sigma_{i}\right)} \end{array}\right)$$ (22)

As in Eq 22, first step for computing the gradient vector is to consider that only *A*_*i*_ varies, while keeping *σ*_*i*_ fixed. Δ*A* is chosen as a step that will vary the amplitude. We have experimentally selected Δ*A* = 0.01 and Δ*σ = pi*/18. In the second case only *σ*_*i*_ varies, while *A*_*i*_ is considered constant (Table 1).

**Table 1:** Gradient algorithm.

/* Initialization */
1 Δ*A* = constant step
2 Δ*σ* = constant step
3 $\psi \;=\;\left[\psi_{A_{i}};\psi_{\sigma_{i}}\right]$
/* Step 1—amplitude varies while phase is fixed */
4 *A*_*i*_(t+1) = *A*_*i*_(t) + Δ*A*
5 *σ*_*i*_(*t*+1) = *σ*_*i*_(*t*)
6 $\hat{y}(t)\;=\;Model(u,A_{i}(t+1),\sigma_{i}(t+1))$
7 Δ*y = Model*(*u*,*A*_*i*_(t+1),*σ*_*i*_(*t*+1))−*Model*(*u*,*A*_*i*_(t),*σ*_*i*_(*t*))
8 $\psi_{A_{i}}\;=\;\frac{\Delta y}{\Delta A}$
/* Step 2—phase varies while amplitude is fixed */
9 *A*_*i*_(t+1) = *A*_*i*_(t)
10 *σ*_*i*_(*t*+1) = *σ*_*i*_(*t*) + Δ*σ*
11 $\hat{y}(t)\;=\;Model(u,A_{i}(t+1),\sigma_{i}(t+1))$
12 Δ*y = Model*(*u*,*A*_*i*_(t+1),*σ*_*i*_(*t*+1))−*Model*(*u*,*A*_*i*_(t),*σ*_*i*_(*t*))
13 $\psi_{\sigma_{i}}\;=\;\frac{\Delta y}{\Delta \sigma }$

### 6.3. Algorithm evaluation

**Fig 12:**
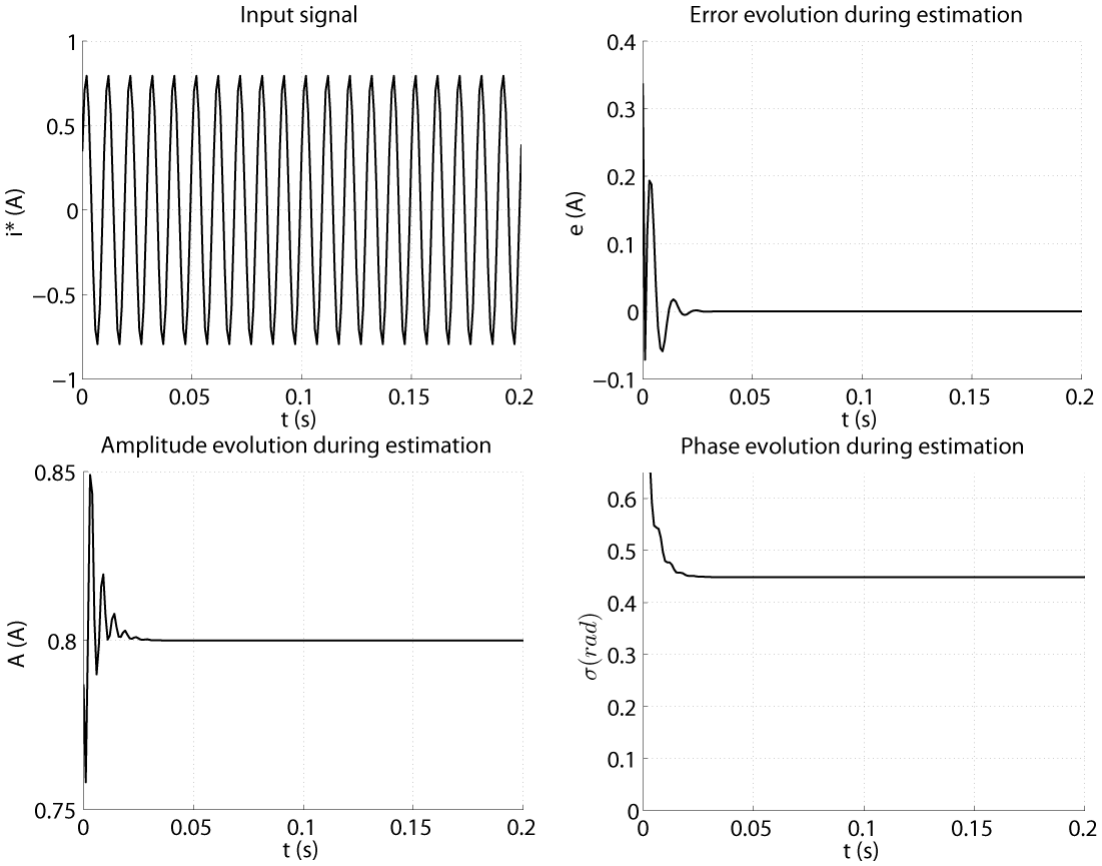
Offline parameters estimation graphs.

The algorithm from Table 1 is evaluated first off-line, for an ideal case when input signal is a pure sinusoid, with no noise or additional oscillations. Fig 12 shows the evolution of the estimation process. The amplitude and phase are reaching convergent values after approximately 0.1 seconds. The error also drops to zero in about 0.1 seconds.

**Fig 13:**
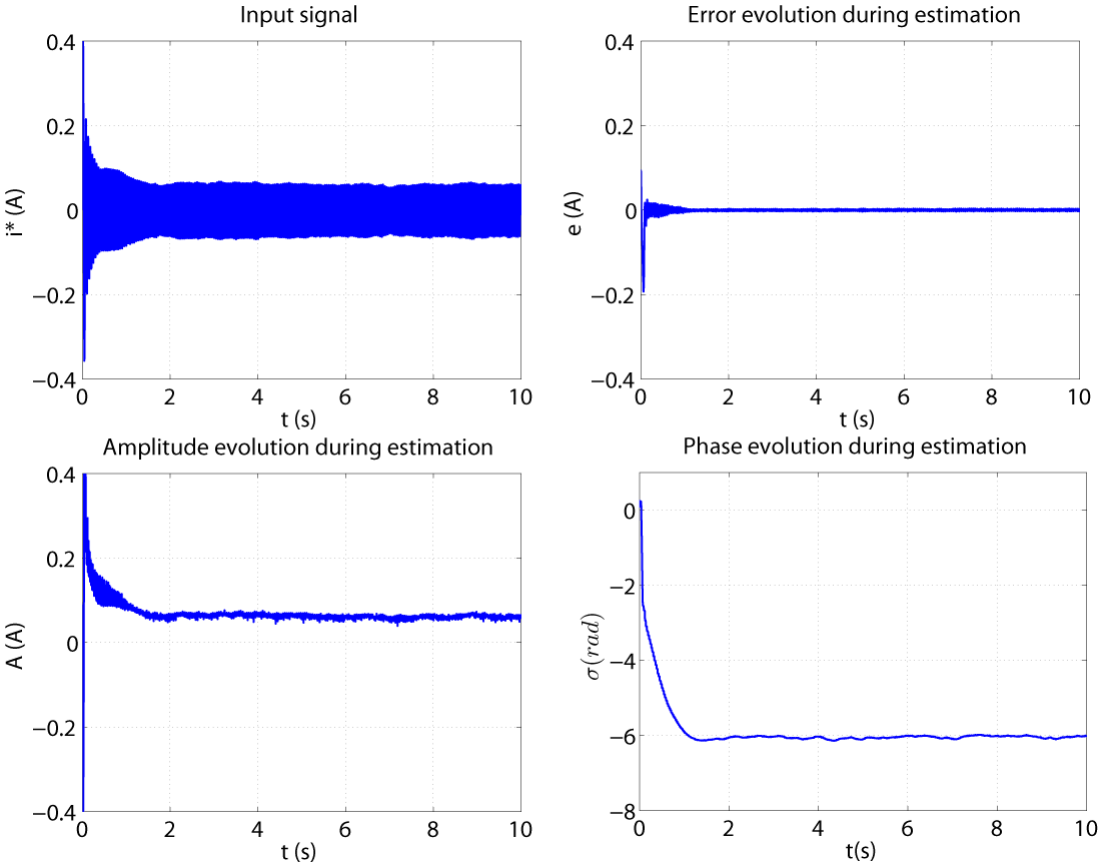
Online parameters estimation graphs for one filtered harmonic.

After assessing the offline results, the online algorithm is evaluated (see S4 and S5 Files). The input signal is the filtered harmonic from the real motor current. Estimation during a time length of 10 seconds is presented in Fig 13. The estimated amplitude and phase are reaching a steady state in about 1.5 seconds. This happens because of the input signal shape, which in the beginning is decreasing (due to the step of current). The error goes towards zero but never reaches it, because of the noisy input signal. Amplitude and phase are presenting some oscillations during estimation, due to the observation error which never gets to zero value. In noisy conditions, an update of the parameters is present in every iteration, thus explaining the ripples in parameter estimation graphs. Most importantly, the phase must have as little oscillations as possible, because a precise estimated phase is needed to avoid any shifting in the compensation process.

### 6.4. On-line results—parameter estimation

**Fig 14:**
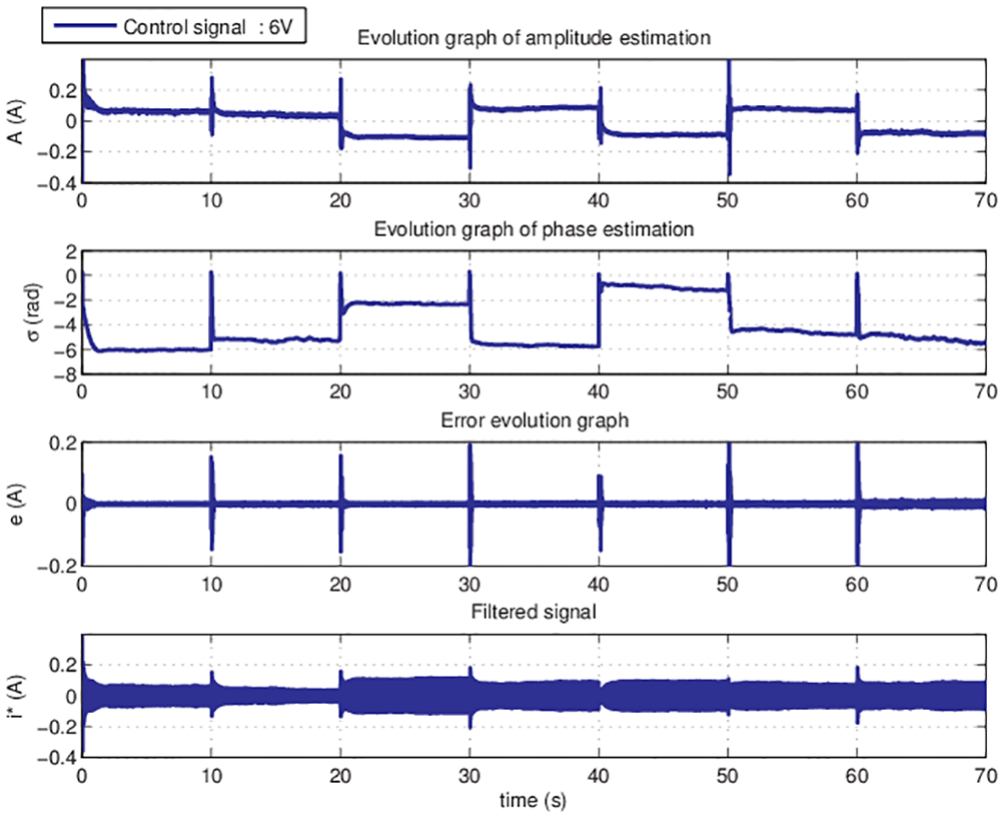
Parameters estimation graph for entire set of harmonics for 6V.

In the Fig 14 are presented online estimation results for the entire set of considered harmonics, for a 6V control signal input.

The estimation of the parameters for each selected harmonic is done for 10 seconds. The estimated values are stored in a memory block from where they are read by the periodic disturbances model, for creating the compensation signal.

The factor *λ* is tested for different values. Due to constant noise present in the input signal, this parameter needs to be tuned for finding a compromise value in order to achieve good results in estimation process for all considered harmonics. *λ* is tested for values in interval 0.05–0.4. The amplitude and phase are converging for *λ* values inside this range. The quality of estimation is low, if some harmonics have a slow convergence (or no convergence at all). Different *λ* values with a 0.02 step are tested. It has been concluded that for *λ* = 0.1, good estimation results are obtained for all considered harmonics.

## Experimental Results

In this section, the control loop is evaluated on the experimental test bed under various excitation signals, using the built controller. The implementation procedure includes following steps:

- Simulink implementation is loaded in Simulink framework and compiled.
- System target file is chosen which defines the process of generating C code for Real-Time Windows Target.
- After the compilation is done with no error and the value for the control signal is selected, the evaluation can begin.

Model parameters are estimated during 10 seconds for each harmonic and values are stored in a memory block. At the end of the estimation process, the periodic disturbances model reads the parameters estimated for each harmonic from the memory block and shapes the compensation signal. This signal is added to the input excitation signal of the DC servo motor.

After the period of 70 seconds needed for filtering and estimation the compensation is automatically turned on. Current spectra, before and after the compensation are compared to reveal the results. Results from each stage of the implementation (i.e. filter output) are also plotted for each evaluation.

**Fig 15:**
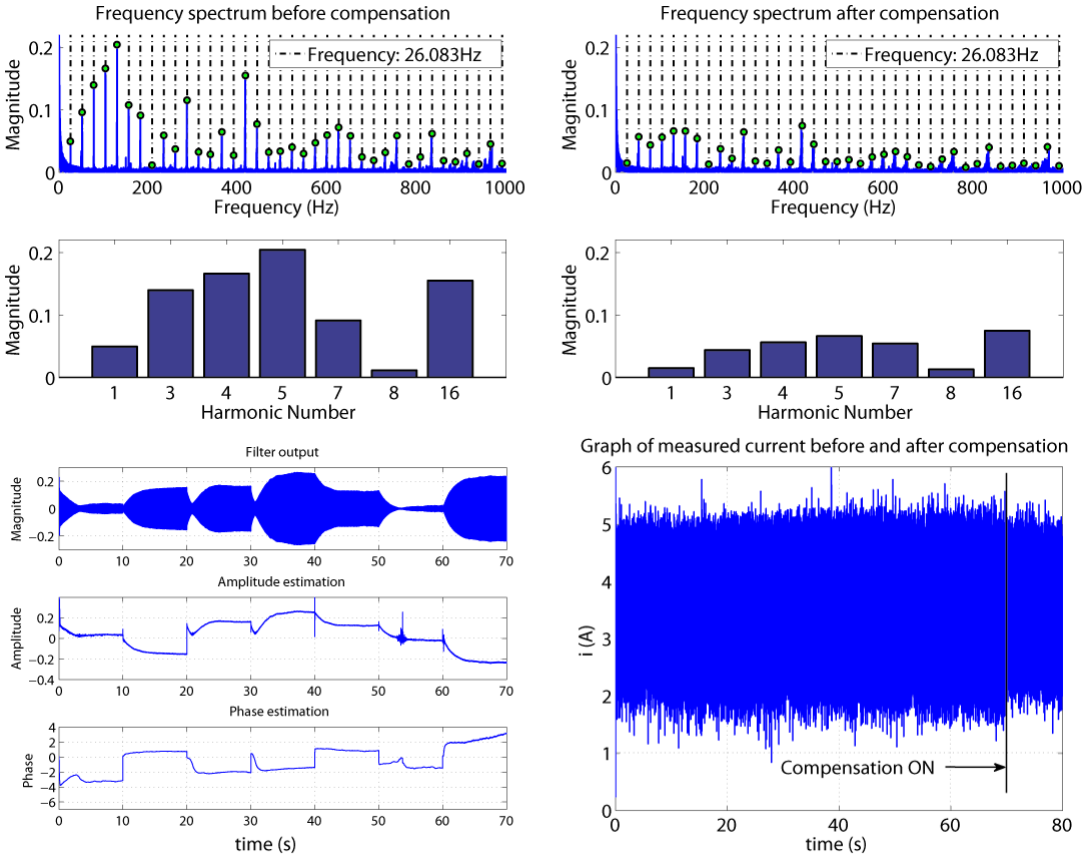
Compensation of a 7V excitation signal with no load.

In Fig 15, considering an excitation signal of 7V (arbitrary chosen), frequency spectra of the current are compared before and after the compensation. Here it can be seen that harmonics 1, 3, 4, 5, 7, 8, 16 are suppressed without inserting any additional noise.

Filter output and evolution of model parameters estimation for each harmonic are also plotted. The last graph represents the current signal before and after the compensation is turned on.

**Fig 16:**
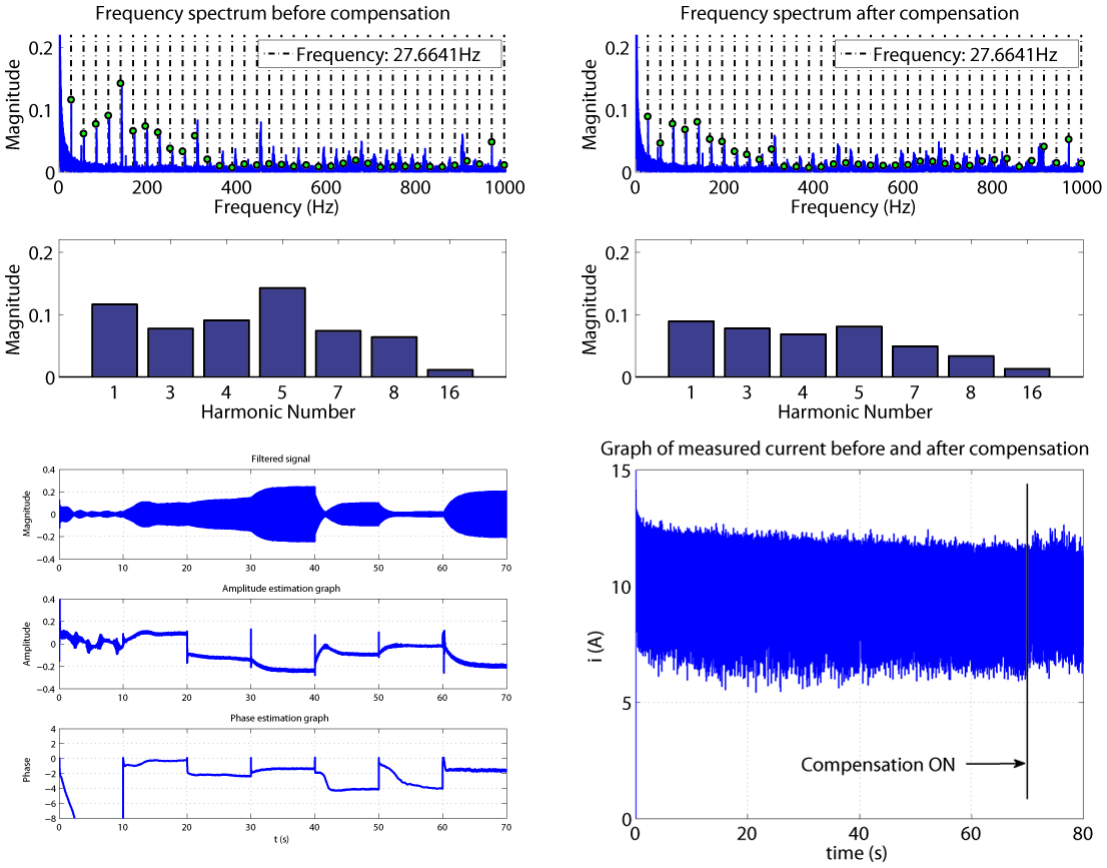
Compensation of a 7V excitation signal with load.

The controller is evaluated also with applied load by coupling the motor shaft with the gear box (Fig 16). In this case, the harmonics are decreased, but with a smaller amount than in the case of compensation without load.

## Conclusions

In this work, periodic disturbances inside PM motors are identified and compensated. Detection of the periodical disturbances is done based on a systematic analysis of the collected data from the real system in frequency domain.

For the online identification of model parameters, an adaptive filter is implemented and a recursive algorithm based on normalized gradient is chosen. The implementation of the control loop is running in real time, thus only embedded functions and Simulink standard blocks were used. The implementation allows easy parameter customization (i.e. the considered harmonics and the actualization gain for the observation error). Even if the proposed method presents difficulties in noisy spectrum, it is shown that through an optimized algorithm, it is possible to compensate the considered harmonics, thus reducing vibration and noise.

**Fig 17:**
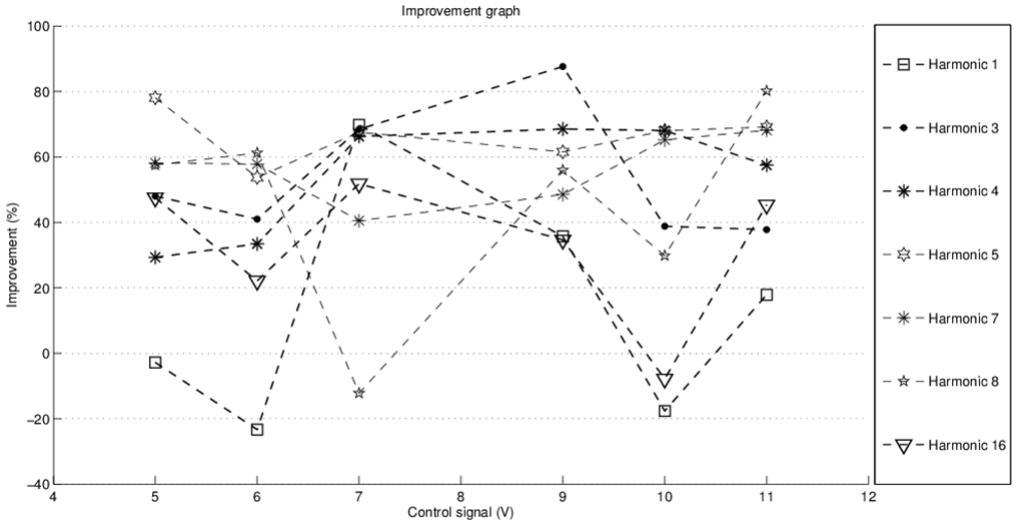
Improvement graph.

The relative amount of compensation improvement is presented in Fig 17. Each particular harmonic is analyzed over 5V-11V range. A good compensation is obtained when the control signal doesn’t reach values close to the borders of the operation range. Between 7V-10V, the compensation has good results, decreasing the considered harmonics. Compensation is ineffective for some of the cases, because the estimated parameters are not reaching the limit values, due to the oscillations of filtered harmonics.

## Supporting Information

S1 Files Measurements data acquisition from DC motor. Contains 2 m files for plotting and a dataset recorded from the motor.
(ZIP)

S2 Files Offline analysis transform to frequency domain and grid. Contains the frequency domain analysis and the harmonic computation m files, together with a dataset recorded from the motor.
(ZIP)

S3 Files Plot distribution of harmonics. Contains the m files for plotting the harmonics distribution, together with a load input dataset.
(ZIP)

S4 Files MATLAB model—Simulink estimation model. (ZIP)

S5 Files MATLAB model—Simulink online observer model. (ZIP)

S6 Files MATLAB model—Simulink offline observer model and input dataset. (ZIP)
